# Do motivations for using Facebook moderate the association between Facebook use and psychological well-being?

**DOI:** 10.3389/fpsyg.2015.00771

**Published:** 2015-06-12

**Authors:** James R. Rae, Susan D. Lonborg

**Affiliations:** ^1^Department of Psychology, University of Washington, SeattleWA, USA; ^2^Central Washington University, EllensburgWA, USA

**Keywords:** Facebook, media consumption, motivations, psychological well-being

## Abstract

Previous investigations of the relationship between Facebook use and psychological well-being have most commonly considered variables relating to the *quantity* (e.g., time spent online) and *underlying motivations* (e.g., making new friends) of Facebook consumption. However, previous research has reached contradictory conclusions in that quantity of Facebook use has been linked to both higher and lower levels of psychological well-being. The current study investigated whether these contradictory findings of quantity of Facebook use could be explained by considering users’ motivations for accessing Facebook. We predicted that quantity of use would be positively associated with psychological well-being when users primarily accessed Facebook to maintain existing relationships but negatively associated with psychological well-being when primarily accessed to create new relationships. In a sample of college undergraduates (*N* = 119), we found that the relationship of quantity of Facebook use on psychological well-being was moderated by the motivation of the user. Quantity of Facebook use was associated with higher levels of psychological well-being among users that accessed Facebook for friendship purposes but was negatively associated with psychological well-being among users that accessed Facebook for connection purposes (e.g., making new friends). We also replicated our results across dimensions of psychological well-being (e.g., anxiety and life satisfaction). The current findings provide initial evidence that quantity and motivations of Facebook use interact with potentially serious implications for psychological well-being and also provide a possible explanation for why quantity of Facebook use can be linked with both positive and negative psychological well-being.

## Introduction

Facebook is the largest social networking site in the world with over 1.35 billion active monthly users (Facebook Press Room, 2014), and given the widespread use and popularity of the site, researchers have recently recognized the need to understand how Facebook use impacts users’ health (Rajani et al., 2011). To this end, at least two distinct approaches have independently investigated how the *quantity* of Facebook use (e.g., number of Facebook friends, time spent using Facebook) or *motives for why* users access Facebook (e.g., making new friends, maintaining ties with current friends) relate to psychological well-being in samples of college students (among the most intense users of Facebook; Jacobsen and Forste, 2011; Kalpidou et al., 2011). Similar to findings that suggest that while the Internet is often used for social interaction, Internet use can often result in increased loneliness and depression (termed the “Internet paradox”; Kraut et al., 1998), previous research has found that quantity of Facebook use can be both *positively* and *negatively* associated with psychological well-being. In this research, we investigate whether these contradictory findings considering quantity of Facebook use can be resolved by considering the motives for why users access Facebook.

One line of research has investigated the relationship between Facebook use and psychological well-being by focusing on quantity of Facebook use variables (e.g., number of friends, duration, and frequency of site use). Interestingly, initial findings in this line of research have reached seemingly mixed conclusions: On one hand, research has suggested that “intensity” of Facebook use is positively associated with life satisfaction (Ellison et al., 2007; Valenzuela et al., 2009), both exposure to ones’ own profile (Gonzales and Hancock, 2011) and time spent using Facebook (Gentile et al., 2012) are positively associated with self-esteem, and that the number of Facebook friends is negatively associated with loneliness (Burke et al., 2010). On the other hand, other research has found that both time spent using Facebook (Kalpidou et al., 2011) and the frequency of checking one’s Facebook account (Mehdizadeh, 2010) is associated with lower self-esteem. Further, duration of Facebook use was positively associated with user’s perceptions that others had both better lives and were happier than themselves, as well as lower endorsement of perceptions that life is fair (Chou and Edge, 2012). Like previous research, we present quantity of Facebook use variables as predictors of psychological well-being. However, the associative relationship between these variables may be bi-directional (Sheldon et al., 2011), such that psychological well-being may be predictive of higher quantities of Facebook use.

Other research has investigated the relation between motivations for using Facebook and psychological well-being (Shields and Kane, 2011). For example, uses and gratifications theory postulates that the desires that motivate media consumption are important to consider in understanding mass communication. For uses specific to Facebook, while additional motivations have been occasionally reported (e.g., reputation enhancement; Joinson, 2008), the two primary motives are seeking new relationships or cultivating existing relationships (Lampe et al., 2006; Joinson, 2008; Lenhart et al., 2008; Subrahmanyam et al., 2008; Bonds-Raacke and Raacke, 2010; Tosun, 2012; compare with Nadkarni and Hofmann, 2012). Further, initial evidence suggests that these primary motivations of Facebook use may also be predictive of psychological well-being. For example, incoming college students that used Facebook to develop new connections reported lower social adjustment and higher levels of loneliness while incoming students that used the site to maintain existing relationships were more likely to report better social adjustment and lower levels of loneliness (Yang and Brown, 2013). Similarly, users that reported higher numbers of Facebook friends that they did *not* personally know, indicating a pattern of establishing online connections in the absence of established oﬄine relationships, reported higher endorsement of perceptions that others had better lives than themselves (Chou and Edge, 2012).

In the present study, we ask whether two facets of Facebook use that have been investigated independently (i.e., as main effects), the quantity and motivations for Facebook use, are more informative when considered in tandem (i.e., as an interaction). More specifically, while quantity of Facebook use has been shown to be both positively and negatively related to psychological well-being, we hypothesize that these contradictory results may be resolved in part by considering the underlying motivation of the user: When Facebook use is generally focused toward maintaining existing relationships, we predict quantity of Facebook use will be associated with higher levels of psychological well-being. When Facebook use is generally focused toward establishing new relationships, we predict that quantity of Facebook use will be associated with lower levels of psychological well-being. If our hypothesis of an interaction between quantity of use and motivations for using Facebook is correct, the highest (for those that use the site to primarily maintain existing connections) and lowest (for those that use the site to primarily establish new connections) levels of psychological well-being should be found among those users that have the greatest quantity of use.

## Materials and Methods

### Participants

Participants were undergraduates (*N* = 129) enrolled in psychology courses at a rural Northwest university. Due to experimenter error, three participants did not receive complete study materials and were removed from the analysis. An additional seven participants were removed due to missing responses on the scale used as the criterion variable, which resulted in a total sample of 119 participants. Females comprised 73.6% of the sample, which is consistent with the gender composition of undergraduate psychology majors (Pion et al., 1996). Most participants were between 18–21 years (75.4%) and 22–25 years (17.8%) of age and within the sample there were roughly equal proportions of freshman (19.5%), sophomores (34.8%), juniors (28.0%), and seniors (16.9%)^1^. About half of participants held some form of employment (50.8%) and roughly equal proportions of participants were enrolled part-time (55.2%) and full-time (44.8%). To maintain the anonymity of participants, questions pertaining to race/ethnicity were formatted as open-ended questions and emphasized to be voluntary. Of the 91 (70.5% of total sample) respondents that did report their ethnicity, 85.2% were white, 7.4% identified as Hispanic, and 7.4% identified as multiracial. Being 18 years of age or older was the only eligibility requirement for this study.

### Measures

#### Quantity of Facebook Use

To index quantity of Facebook use, all participants completed two items assessing how often they use Facebook and their number of Facebook friends (Ellison et al., 2007). Although the items may be formatted as either open-ended or closed-ended questions, for simplicity in scoring, these items were formatted as closed-ended ordinal items (using intervals from previous research): To measure number of Facebook friends, a nine-point scale (1 = 10 or fewer; 9 = More than 400) was used and to measure time spent per day using Facebook, a six-point scale was used (1 = Less than 10 min; 6 = More than 3 h).

#### Motivations for Facebook Use

Participants’ motivations for using Facebook were measured using 11 self-report items (**Table 1**) that were developed with college undergraduate samples (Raacke and Bonds-Raacke, 2008; Bonds-Raacke and Raacke, 2010). Items were scored using a seven point Likert-scale (1 = does not apply to me at all to 7 = definitely applies to me) according to how well that motive exemplified their Facebook usage. A previously reported (Bonds-Raacke and Raacke, 2010) factor analysis of the reasons for Facebook use items produced three overarching dimensions: information, friendship, and connection (see **Table 1**).

**Table 1:** Items loading onto dimensions of Facebook use.

Reasons for Facebook use dimensions
**Dimension 1: information**
(1) To post social functions
(2) To learn about events
(3) To share information about yourself
(4) For academic purposes
(5) To post/look at pictures
**Dimension 2: friendship**
(1) To keep in touch with old friends
(2) To keep in touch with current friends
(3) To locate old friends
**Dimension 3: connection**
(1) For dating purposes
(2) To make new friends
(3) To feel connected

#### Psychological Well-Being

Psychological well-being was measured using the Mental Health Inventory, which is a 38-item self-administered instrument that assesses participant’s self-reported thoughts and feelings within the past month (Veit and Ware, 1983). Individual items correspond to six mental health constructs that formulate two subscales: psychological distress (anxiety, depression, loss of behavioral/emotional control) and psychological well-being (general positive affect, life-satisfaction, emotional ties). The two subscales may also be aggregated into an overall global health factor, the Mental Health Index (hereafter, scores on the MHI refer to the Mental Health Index), by scoring each item so that higher scores indicate more frequent occurrence of positive mental health symptoms (or less frequent occurrence of negative mental health symptoms). As such, higher scores on the MHI indicate greater psychological well-being. Although prior research has evaluated the impact of Facebook use on specific mental health constructs (e.g., life-satisfaction), to maximize the generalizability of the current study, in the main analyses we elected to use the MHI as a broad measure of psychological well-being that would not be susceptible to nuances of any one construct. Also, previous research use of the MHI with adolescent samples has demonstrated high internal consistency (α = 0.94; Fischer and Shaw, 1999) and high 10-weeks test–retest reliability (*r* = 0.73; Heubeck and Neill, 2000).

### Procedure

The Institutional Review Board granted approval to this study. Participants were recruited for study participation through an online website that facilitates psychological research at the university. Once registered for the study, potential participants were redirected to the online survey service Qualtrics where they were presented with the IRB approved information sheet that contained the contact information of both authors, study information, and participant rights as well as the survey items for this study. Prior to completing any study materials, all participants indicated that they understood the risks and wished to participate in the research. Participation was anonymous; as such, no identifying information was collected and credit was automatically awarded at the completion of the survey. Course credit was awarded for participation.

### Analytic Method

To obtain indicators for users’ motivations for accessing Facebook, we first computed subscale scores by computing the means of all items that loaded onto the three dimensions of Facebook use (hereafter referred to as information, friendship, and connection; see **Table 1** for factors and items) identified in previous research (Bonds-Raacke and Raacke, 2010). Drawing on previous research suggesting that motivations for accessing Facebook are associated with psychological well-being, we predicted that connection, which most closely parallels the motivation of seeking new connections, would be *negatively* related to scores on the MHI. Conversely, we predicted that friendship, which most closely parallels maintaining current connections, would be *positively* related to scores on the MHI (Yang and Brown, 2013). We made no specific prediction regarding information. As measures of quantity of Facebook use, we included time spent using Facebook per day and number of Facebook friends, but we did not make any directional predictions regarding these variables. We also controlled for time spent using the Internet for non-academic purposes per week^2^. Central to the hypothesis tested in this research, we predicted a positive interaction between friendship purposes and quantity of Facebook use variables and a negative interaction between connection purposes and quantity of use variables. Lastly, due to recent concerns about the selective use of covariates in psychological research (Simmons et al., 2011), we first report the results of the main effects and interactions between the motivations for Facebook use subscales and quantity of use without including control variables. Next, we added covariates into the models and re-examined the original pattern of results.

## Results

### Preliminary Analyses

See **Table 2** for means, standard deviations (SD), and reliability estimates of study measures. See **Table 3** for zero-order correlations among predictor variables.

**Table 2:** Mean, SD, and reliability index for study measures.

Measures (subscale in italics)	Mean	SD	Range		α
			Minimum	Maximum	
Facebook friends	7.12	2.02	1.00	9.00	-
Time using Facebook	3.01	1.37	1.00	6.00	-
*Information*	3.80	1.27	1.00	6.60	0.73
*Friendship*	5.05	1.29	1.00	7.00	0.63
*Connection*	2.96	1.28	1.00	7.00	0.62
Psychological well-being	161.1	27.90	91.00	210.00	0.94
*Anxiety*	24.78	7.51	12.00	47.00	0.88
*Depression*	10.03	3.72	4.00	20.00	0.86
*Loss of control*	20.06	6.98	10.00	39.00	0.85
*Positive affect*	38.63	7.85	21.00	56.00	0.89
*Life satisfaction*	4.26	1.00	2.00	6.00	-
*Emotional ties*	8.63	2.67	2.00	12.00	0.79

Reliability estimates for Facebook friends, Time using Facebook, and Life satisfaction are not presented as they were indexed with single items.

**Table 3:** Zero-order correlations among variables predicting psychological well-being.

	Predictor	1	2	3	4	5	6	7	8	9
(1)	Information	1								
(2)	Connection	0.58	1							
(3)	Friendship	0.51	0.33	1						
(4)	Facebook friends	0.19	0.22	0.33	1					
(5)	Time using Facebook	0.25	0.27	0.38	0.29	1				
(6)	Time using Internet	0.02	0.08	0.02	-0.01	0.19	1			
(7)	Year in school	-0.03	-0.02	0.07	0.03	-0.07	0.01	1		
(8)	Gender	0.11	0.00	0.04	-0.13	0.07	-0.14	-0.14	1	
(9)	Course credits taken	0.00	-0.04	0.02	0.03	-0.06	-0.11	-0.03	-0.06	1

Gender was dummy coded (0 = Female, 1 = Male).

### Regression Analyses

To investigate the relationship between quantity/duration of Facebook use and motivations for using Facebook on psychological well-being, we conducted a hierarchical regression analysis using MHI as the criterion variable. In Step 1, we included information, friendship, connection, time spent using Facebook, and Facebook friends as predictors (all mean-centered). Number of Facebook friends was the only significant predictor of scores on the MHI, such that participants that had reported having more Facebook friends tended to report higher levels of psychological well-being (β = 0.25, *p* = 0.010; see **Table 4**)^3^.

**Table 4:** Summary of regression analyses for Facebook variables predicting Mental Health Index (MHI) scores.

	Step 1			Step 2			Step 3		
Variable	*B*	SE B	β	*B*	SE B	β	*B*	SE B	β
Information	-1.42	2.64	-0.06	-0.97	2.67	-0.04	-1.80	2.77	-0.11
Friendship	4.58	2.42	0.21^†^	4.44	2.50	0.21^†^	4.75	2.55	0.23^†^
Connection	-2.85	2.42	-0.13	-2.48	2.43	-0.11	-1.61	2.52	-0.06
Facebook time	-2.41	2.02	-0.12	-2.80	2.03	-0.14	-2.39	2.13	-0.12
Facebook friends	3.53	1.34	0.25^∗^	4.06	1.43	0.29^∗∗^	3.52	1.52	0.28^∗^
Time^∗^information				1.27	1.78	0.08	1.41	1.83	0.08
Time^∗^friendship				2.57	1.70	0.16	2.56	1.74	0.16
Time^∗^connection				-4.10	1.86	-0.26^∗^	-4.32	1.97	-0.27^∗^
Friends^∗^information				-0.48	1.49	-0.04	-0.29	1.58	-0.02
Friends^∗^friendship				-0.39	1.07	-0.04	-0.46	1.12	-0.04
Friends^∗^connection				1.72	1.40	0.16	1.29	1.55	0.09
Year in school							-1.09	2.57	-0.09
Internet							-0.30	0.29	-0.10
Gender							0.74	6.32	0.10
Course credits							0.92	4.07	0.06
*R^2^*	0.11			0.17			0.17		
Change in *R^2^*				0.06			0.00		

Continuous predictors centered at their means (*B*) and standardized (β). Covariates were categorical and not mean centered or standardized in any models. ^∗^*p* < 0.05, ^∗∗^*p* < 0.01, ^†^*p* < 0.10.

**FIGURE 1:**
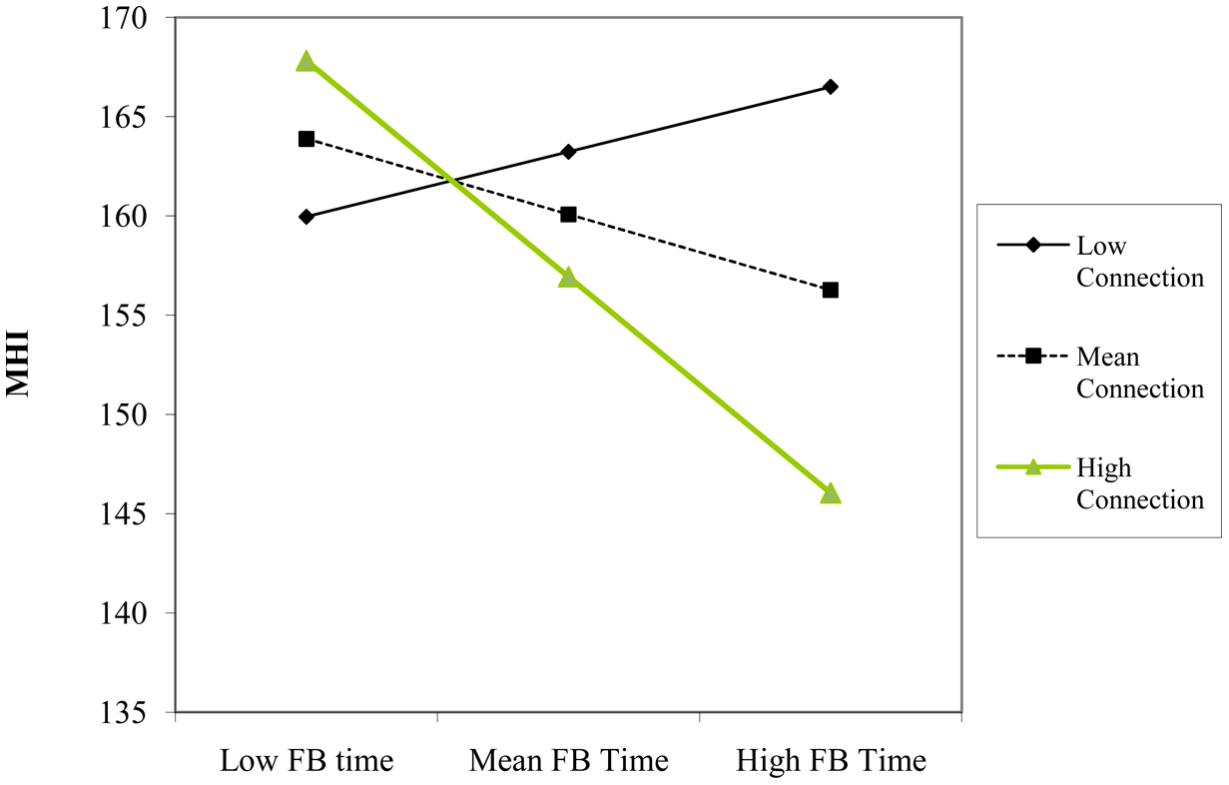
**Participant’s Facebook time and connection level as interactive predictors of overall scores on the Mental Health Index (MHI)**.

In Step 2, number of Facebook friends (β = 0.29, *p* = 0.005) remained a significant predictor of scores on the MHI even after adding the two-way interactions between motivations for Facebook use and quantity of Facebook use variables. There was not a significant increase variance explained by including the two-way interactions between Facebook use variables, *F*(6,105) = 1.21, *p* = 0.309. As predicted, there was a significant two-way interaction between time spent using Facebook and using Facebook for connection purposes (β = -0.26, *p* = 0.030). Simple slopes analyses indicated that time spent using Facebook was not associated with scores on the MHI at one SD below the mean of connection (β = 0.11, *p* = 0.431), but was a significant predictor at one SD above the mean of connection (β = -0.39, *p* = 0.017; see **Figure 1**). Therefore, consistent with our hypothesis, time spent using Facebook was *negatively* associated with psychological well-being only for those participants that reported higher connection motivations driving their Facebook use.

In Step 3, we adjusted for year in school, time spent using the Internet for non-academic purposes, gender, and course credits^4^. Other than number of Facebook friends (β = 0.28, *p* = 0.022) and the two-way interaction between time spent using Facebook and connection purposes (β = -0.27, *p* = 0.030), none of the covariates, Internet, or Facebook use variables (main effects and interactions) were associated with scores on the MHI. There was not a significant increase variance explained by including covariates into the model, *F*(4,98) = 0.48, *p* = 0.75. To assess multicollinearity among predictor variables in Step 3, we computed a variance inflation factor (VIF) for each predictor variable. The largest VIF was 2.96, which falls well below the rule of thumb for indicating high multicollinearity (10; Kutner et al., 2004).

### MHI Subscales

While the aforementioned analyses provide some evidence of an interaction between Facebook motivations and quantity of Facebook use, it could be possible, and perhaps even likely, that the patterns of results reported above vary across all the constructs that formulate the two MHI subscales of psychological well-being (general positive affect, emotional ties, and life satisfaction) and psychological distress (loss of behavioral/emotional control, anxiety, and depression). Therefore, we tested this possibility by computing separate regression models using each construct score of the MHI as a separate criterion variable. However, instead of using the entire hierarchical regression testing procedure for each individual construct of the MHI (6 constructs × 3 Steps = 18 models), we chose to reduce the number of models considered by only fitting one model for each construct using all the predictors included in Step 3 in the aforementioned analyses (see **Table 4**).

#### General Positive Affect

We found that using Facebook for friendship purposes (β = 0.24, *p* = 0.036) and number of Facebook friends (β = 0.26, *p* = 0.018) predicted *higher* levels of general positive affect. There were no significant interactions between Facebook use variables.

#### Emotional Ties

Using emotional ties as the criterion variable, we found no statistically significant main effects or interactions.

#### Life Satisfaction

**FIGURE 2:**
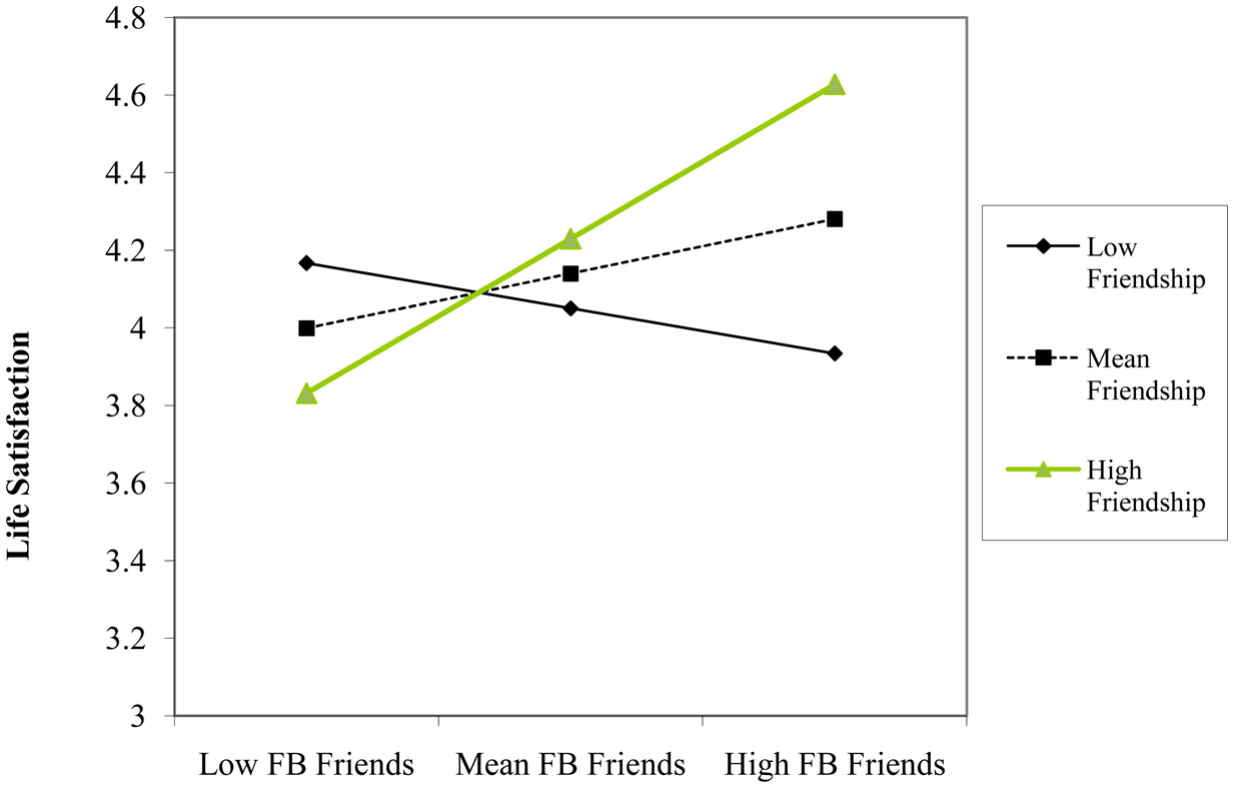
**Participant’s number of Facebook friends and friendship level as interactive predictors of scores on life satisfaction construct**.

Using life satisfaction as the criterion variable, we found no statistically significant main effects of Facebook use variables or covariates. However, there was a significant two-way interaction between using Facebook for friendship purposes and number of Facebook friends (β = 0.25, *p* = 0.021). Simple slopes analyses indicated that number of Facebook friends was not a significant predictor of life satisfaction at one SD below the mean of friendship purposes (β = -0.13, *p* = 0.344), but was a significant predictor at one SD above the mean of friendship purposes (β = 0.36, *p* = 0.030). That is, number of Facebook friends predicted *higher* levels of life satisfaction, but only among those participants that indicated *higher* levels of friendship motivations for accessing Facebook (see **Figure 2**).

#### Loss of Behavioral/Emotional Control

**FIGURE 3:**
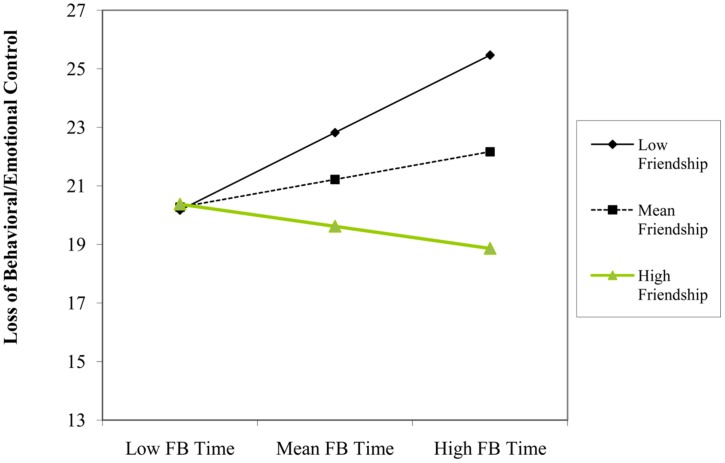
**Participant’s Facebook time and friendship level as interactive predictors of scores on loss of behavioral/emotional control construct**.

**FIGURE 4:**
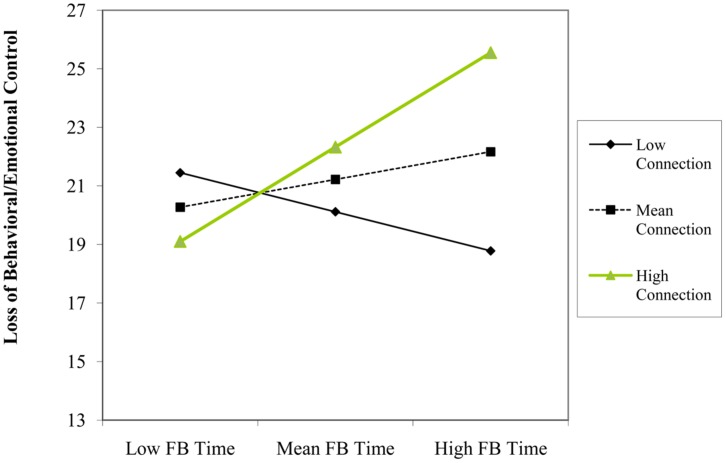
**Participant’s Facebook time and connection level as interactive predictors of scores on loss of behavioral/emotional control construct**.

**FIGURE 5:**
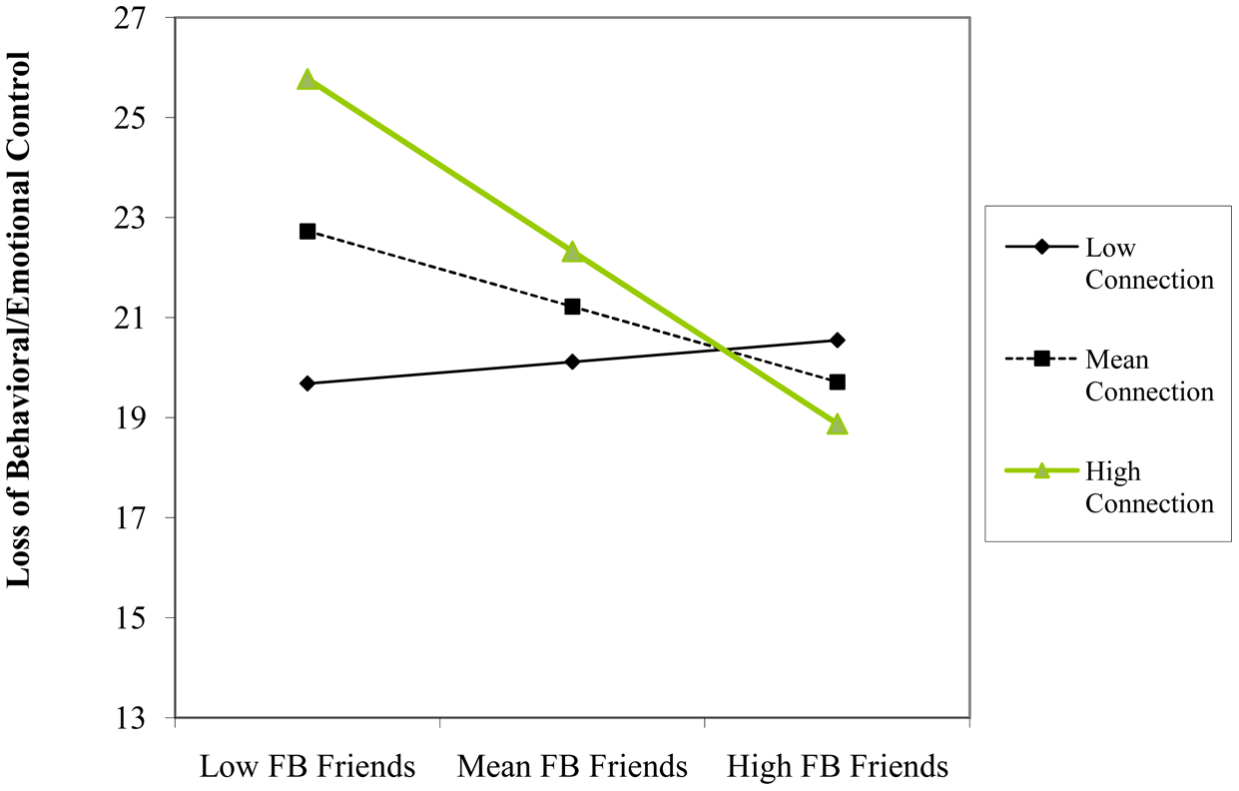
**Participant’s number of Facebook friends and connection level as interactive predictors of scores on loss of behavioral/emotional control construct**.

We found that using Facebook for friendship purposes (β = -0.23, *p* = 0.046) and number of Facebook friends (β = -0.22, *p* = 0.045) predicted *lower* levels of loss of behavioral/emotional control. Although there was not a significant main effect of time spent using Facebook on loss of behavioral/emotional control, this variable significantly interacted with both using Facebook for friendship (β = -0.24, *p* = 0.025) and connection (β = 0.33, *p* = 0.008) motivations, but with different consequences. Simple slopes indicated that time spent using Facebook predicted *higher* levels of loss of behavioral/emotional control at one SD below the mean of friendship (β = 0.38, *p* = 0.015), but was non-significant at one SD above the mean (β = -0.10, *p* = 0.492; see **Figure 3**). Further, time spent using Facebook predicted *greater* levels of loss of behavioral/emotional control at one SD above the mean of connection (β = 0.35, *p* = 0.003), but not at one SD below the mean (β = -0.14, *p* = 0.230; see **Figure 4**). We also found a two-way interaction between number of Facebook friends and using Facebook for connection purposes (β = -0.28, *p* = 0.048). However, contrary to our hypothesis that quantity of Facebook use would have adverse mental health consequences (e.g., higher loss of behavioral/emotional control) for users that accessed the site for connection purposes, simple slopes analyses indicated that while number of Facebook friends did not predict loss of behavioral/emotional control at one SD below the mean of connection (β = 0.04, *p* = 0.818), it was a *negative* predictor at one SD above the mean of connection (β = -0.49, *p* = 0.012; see **Figure 5**).

#### Depression

**FIGURE 6:**
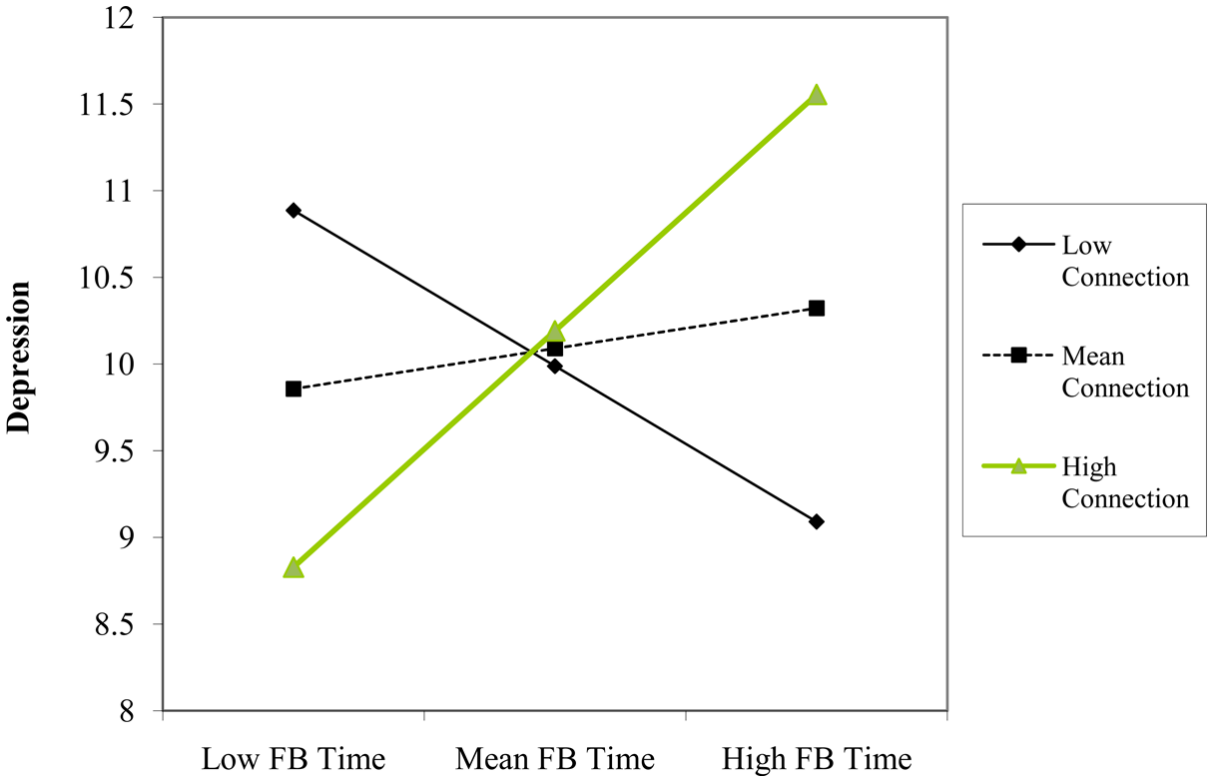
**Participant’s Facebook time and connection level as interactive predictors of scores on depression construct**.

Using depression as the criterion variable, we found that number of Facebook friends predicted lower levels of depression (β = -0.22, *p* = 0.048). There was also a significant interaction between using Facebook for connection reasons and time spent using Facebook (β = 0.35, *p* = 0.007). Simple slopes analyses indicated that time spent using Facebook was not a significant predictor of depression at one SD below the mean of connection purposes (β = -0.31, *p* = 0.066), but was a significant predictor at one SD above the mean (β = 0.40, *p* = 0.015). That is, time spent using Facebook predicted *higher* levels of depression, but only among the users that most frequently accessed the site for connection reasons (see **Figure 6**).

#### Anxiety

**FIGURE 7:**
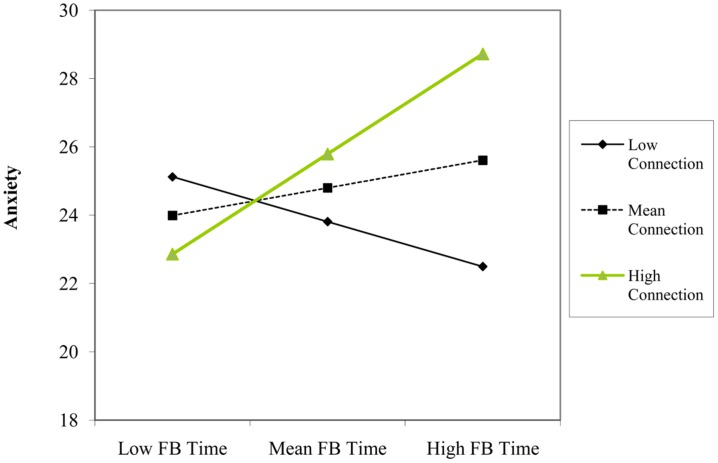
**Participant’s Facebook time and connection level as interactive predictors of scores on anxiety construct**.

Using anxiety as the criterion variable, we found no statistically significant main effects of Facebook use variables or covariates. There was a significant two-way interaction between using Facebook for connection purposes and time spent using Facebook (β = 0.30, *p* = 0.022). Simple slopes analyses indicated that time spent using Facebook was not a significant predictor of anxiety at one SD below the mean of connection purposes (β = -0.20, *p* = 0.234), but was a significant predictor at one SD above the mean (β = 0.42, *p* = 0.013). That is, time spent using Facebook predicted *higher* levels of anxiety, but only among the users that most frequently accessed the site for connection reasons (see **Figure 7**).

## Discussion

In the present research, we investigated whether the previously reported contradictory effects of quantity/duration of Facebook use, which have been both positively and negatively related to psychological well-being in prior research (Boyd and Ellison, 2007; Chou and Edge, 2012), may be explained by considering the moderator of user motivation (Katz et al., 1974). In our primary analysis, we found support for this hypothesis in that there was a significant interaction between time spent using Facebook and using Facebook for connection reasons. That is, time spent using Facebook was associated with *lower* levels of general psychological well-being among those participants that indicated that connection purposes highly exemplified their Facebook use. Critically, our primary results using the MHI held after controlling for a number of confound variables (e.g., time spent using the Internet). Additional analyses conducted for each construct of the MHI provided further evidence of an interaction between quantity and motivations of Facebook on psychological well-being. As predicted, these effects were limited to social motivations (e.g., connection and friendship). For example, we found that friendship motivations moderated the effect of Facebook friends on life satisfaction such that number of Facebook friends was positively associated with life satisfaction when Facebook use is motivated by a desire to maintain existing relationships.

Further, we also found that connection robustly moderated the effect of Facebook time on each of the three psychological distress constructs, such that time spent using Facebook was associated with higher levels of anxiety, depression, and loss of behavioral/emotional control when connection was a primary motivation of the users. Similarly, time spent using Facebook predicted higher levels of loss of behavioral/emotional control for users whose Facebook use was not motivated by friendship purposes.

Although hypothesized to be statistically significant, the interaction between Facebook friends and connection on loss of behavioral/emotional control was opposite in sign to our prediction. That is, we found that connection moderated the effect of Facebook friends such that more Facebook friends predicted *lower* levels of loss of behavioral/emotional control for those users that accessed Facebook with connection motivations. Perhaps unsurprising in retrospect, it appears that having many Facebook friends is preferable to having few for those users accessing Facebook to establish new relationships or to feel connected (see items for connection factor in **Table 1**). Another unexpected finding was a significant interaction between time spent using Facebook and loss of behavioral/emotional control, such that time spent using Facebook predicted *higher* levels of loss of behavioral/emotional control for users that tended not to access the site for friendship purposes. While we predicted that only connection motivations would moderate any negative effects of quantity of Facebook use on psychological well-being, we do not consider this finding to undermine the support for our hypothesis. Rather, we take both of these results to support our overall conclusion that quantity of Facebook use can be beneficial when used to enhance exchange and engagement within existing relationships and harmful when used for purposes that do not facilitate social engagement or that are aimed at obtaining new connections, perhaps due to difficulty in obtaining oﬄine relationships (Yang and Brown, 2013).

One possible interpretation of the results of the primary and secondary analyses is that class standing, and not motivations for using Facebook, moderate the effects of Facebook use on psychological well-being. That is, it could be possible that time spent using Facebook for connection purposes might particularly exemplify the Facebook use of incoming college students that are both seeking to establish new social connections, and coping with challenges as a first year college student (e.g., living away from home). However, we failed to find evidence for this hypothesis such that we controlled for year in school in our statistical analyses. Another possible interpretation is that the size and daily interaction with oﬄine social networks, and not motivations for Facebook use *per se*, moderate the effects of Facebook use on psychological well-being. By using measured variables in our data set as proxies for oﬄine social network size (number of Facebook friends) and daily interactions (living situation) we failed to find evidence supporting this hypothesis, such that neither of these variables were related to using Facebook for connection purposes^5^. However, a critical direction for future research will be to test this hypothesis more fully. Indeed, future research seeking to establish motivations for Facebook use as a moderator of Facebook use on well-being should both measure and statistically control for the effects of both number and frequency of interactions with oﬄine friends.

The present research is not without limitations. One clear and important limitation is the homogeneity and size of the current sample, which constricts the generalizability of the current study. Therefore, future research might examine whether the effects reported here replicate across more heterogeneous samples of college students (with regards to age, race, etc.), but also to Facebook users more generally. That is, while most research examining the motivations for Facebook use has utilized undergraduate samples, our research suggests the potential importance of determining if non-undergraduate populations generally report the same motivations for using Facebook and if these motivations interact with quantity of use. Also, given the correlational nature of the data, causal relationships cannot be inferred. In this same vein, although we considered quantity of Facebook use variables as predictors of psychological well-being, previous research has indicated that the relationship between these variables may be bi-directional. Indeed, previous studies on social networking site use have suggested that individual’s psychological states drive their online behaviors (Sheldon et al., 2011). Lastly, although significant predictors of psychological well-being, the proportion of variance explained by Facebook related variables was modest (∼17% of the variance).

Despite these limitations, this study makes an important contribution by demonstrating that quantity of Facebook use interacts with Facebook use motivations to have potentially important effects on psychological well-being (both positive and negative). Moreover, this study also provides initial evidence of a possible explanation for why quantity of Facebook use can have both positive and negative effects on psychological well-being. Importantly, our findings have implications for how clinicians define problematic Facebook use. That is, as the debate continues about how best to conceptualize “cyber addictions” in general, and Facebook “addiction” more specifically, and whether or not they constitute actual psychiatric disorders (e.g., Holmes et al., 2014; Suissa, 2015), we suggest that future research should explore efficacious means of assessing problematic use of Facebook and other social networking sites. Whether or not clinicians agree about the severity of such use, we suggest that they nonetheless routinely assess adolescent and young adult clients’ time spent on social networking sites, as well as their motivations for, and consequences of use. Furthermore, clinicians treating these populations of clients may find it helpful to continually monitor the growing body of literature on the psychological sequalae of problematic Internet use, as well as newly proposed methods for intervening with clients (e.g., Cognitive-Behavior Therapy for Internet Addiction; Young, 2011). Rigorous investigation of these cognitive, behavioral, and harm reduction methods for use with such clients should also be continued (e.g., Winkler et al., 2013).

## Conflict of Interest Statement

The authors declare that the research was conducted in the absence of any commercial or financial relationships that could be construed as a potential conflict of interest.

## Footnotes

1 One post-baccalaureate student participated in the study.

2 Given that Internet use independently predicts measures of psychological well-being, such as increased loneliness (Heubeck and Neill, 2000), we controlled for weekly Internet use in all analyses to isolate the unique effects of Facebook related Internet use.

3 For brevity, see regression tables for standardized regression coefficients (β) and *p*-values for all non-significant predictors.

4 Course credits were measured on a 5-point Likert scale using institution-specific values that ranged from part-time to full-time status.

5 These results are available from the first author.
